# Combination of Balsamin and Flavonoids Induce Apoptotic Effects in Liver and Breast Cancer Cells

**DOI:** 10.3389/fphar.2020.574496

**Published:** 2020-10-28

**Authors:** Parminder K. Ajji, Ken Walder, Munish Puri

**Affiliations:** ^1^ Centre for Chemistry and Biotechnology, Deakin University, Geelong, VIC, Australia; ^2^ Centre for Molecular and Medical Research, School of Medicine, Deakin University, Geelong, VIC, Australia; ^3^ Centre for Marine Bioproducts Development, College of Medicine and Public Health, Flinders University, Adelaide, SA, Australia

**Keywords:** bioactives, naringin, naringenin, quercetin, therapeutics, ribosome inactivating proteins

## Abstract

Flavonoids such as naringenin, quercetin, and naringin are known to exhibit anticancer properties. In this study, we examined the effects of these flavonoids on cell viability and apoptotic pathways of cancer cells, either singly or in combination with the type 1 ribosome inactivating protein, Balsamin. Treatment with flavonoids (naringenin, quercetin, and naringin) plus Balsamin for 48 h reduced HepG2 and MCF-7 cell viability, increased the activation of caspase-3 and -8, and induced apoptosis through up-regulation of pro-apoptotic genes and down-regulation of anti-apoptotic genes. Out of the three flavonoids tested, the Balsamin-Naringenin and Balsamin-Quercetin combinations appeared to be most effective compared to the Balsamin-Naringin combination. Balsamin combined with flavonoids also activated endoplasmic reticulum (ER)-stress–mediated apoptosis in breast cancer (MCF-7) cells, which was not activated by Balsamin treatment alone. These experimental results showed that Balsamin combined with flavonoids can reduce HepG2 and MCF-7 cells viability and induce apoptosis, which could be considered as a promising therapeutic approach to sensitize cells to Balsamin treatment, thereby improving its efficacy in breast or liver cancer therapy.

## Introduction

Flavonoids are group of polyphenolic compounds found in fruits, vegetables, herbs, cereals, and dairy products (Cook and Samman, 1996). Flavonoids as a complementary medicine have attracted the attention of researchers due to their diverse pharmacological properties, including antioxidant, antibacterial, antiviral, antitumor, anti-atherosclerosis, antidiabetic, anti-inflammatory, antithrombogenic, hypolipidemic, and neuroprotective effects (Huang et al., 2016). Such biological diversity of flavonoids appears to be associated with their ability to regulate a number of cell signaling cascades, and could play a pivotal role in human health (Williams et al., 2004).

**Figure 1:**
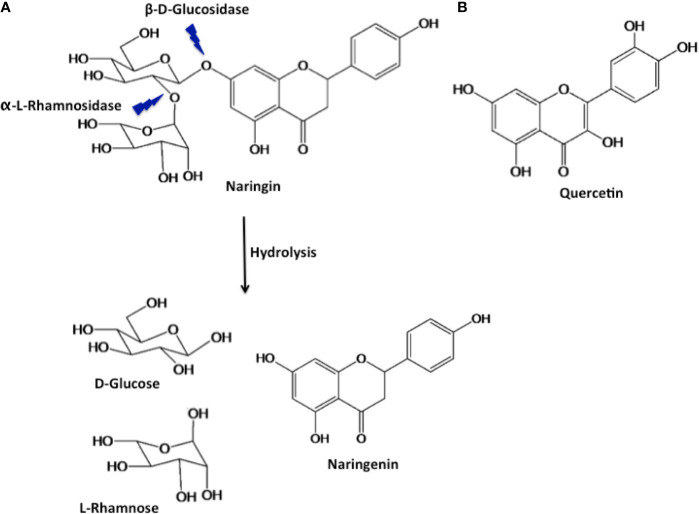
Structure of naringin and its hydrolysed product naringenin **(A)**, and quercetin **(B)**.

Of all the polyphenols known, naringenin (Nar) and its derivatives are known to possess strong antioxidant potential along with other biologically beneficial effects (Salehi et al., 2019). Nar is a flavone, a type of flavonoid that is abundantly found in citrus fruits (Puri et al., 2011). It is derived from the hydrolysis of the glycone form of flavanones, such as naringin (**Figure 1**) (Erlund, 2004). Naringin (Nir) is a flavanone glycoside found in grapes and citrus fruits. It consists of two sugar units (glucose and rhamnose) attached to its aglycon portion, Nar, at the 7-carbon atom (**Figure 1**) (Puri et al., 2012; Alam et al., 2014). Studies have shown that both Nar and Nir exert a variety of common pharmacological effects including antioxidant, anti-inflammatory, anticarcinogenic, and hepatoprotective effects (Bharti et al., 2014). Quercetin (Qu) is a flavonol, another type of flavonoid that is abundantly found in edible fruits and vegetables, and has also gained attention in human health. It consists of two aromatic rings connected via an oxygen containing heterocyclic ring (**Figure 1**) (Gibellini et al., 2011). Qu has reported health benefits similar to Nar and Nir, and it has also been used therapeutically in allergic conditions (such as asthma, hayfever, eczema and hives), arthritis, metabolic syndrome and mood disorders (Kelly, 2011).

Several studies have demonstrated that these flavonoids exhibit anticancer properties toward various cancers, including liver, breast, lung, colon, and bladder cancer (Park and M Pezzuto, 2012; Sharma et al., 2017). Nar inhibited the proliferation of breast cancer (MCF-7) cells by blocking insulin stimulated glucose uptake in GLUT4 (insulin responsive glucose transporter) expressing, insulin responsive MCF-7 cells. Nar blocked glucose uptake by impairing the activation of phosphoinositide3-kinase (P13K), a key regulator of insulin induced GLUT4 translocation, and inhibiting the phosphorylation of p44/p42 mitogen-activated protein kinase (MAPK), a step essential for the insulin signaling pathway (Harmon and Patel, 2004). Nir induced apoptosis in breast cancer cells (MDA-MB-231 and BT549) by increasing the expression of p21 and decreasing the expression of survivin (inhibitor of apoptosis protein, IAP) and active β-catenine (Li et al., 2013). Similarly, Qu supressed the expression of survivin and induced G0/G1 phase cell cycle arrest in breast cancer (MCF-7) cells (Deng et al., 2013). In the case of liver cancer (HepG2) cells, Qu induced apoptosis via inhibition of FASN (fatty acid synthase), a metabolic enzyme that is usually upregulated during early stages of tumorigenesis (Zhao et al., 2014). Nar and Nir induced apoptosis in HepG2 cells via mitochondrial mediated activation of caspase-9 and caspase-8 mediated proteolysis of Bid (Arul and Subramanian, 2013; Banjerdpongchai et al., 2016). This evidence suggests that the anti-cancer effects of these flavonoids involved various mechanisms, a desirable trait in cancer therapeutics.

Balsamin (Bal), a type I ribosome inactivating protein (RIP) from *Momordica balsamina*, exhibits potent anti-HIV activity (Puri et al., 2012). It blocks the replication of HIV by inhibiting the translation step, occurring prior to viral budding and release (Kaur et al., 2013). Our recent studies have shown that Bal exhibits DNase-like activity, and broad spectrum antimicrobial and antioxidant activity (Ajji et al., 2016). Further, in subsequent studies on the anti-proliferative effects of Bal on liver (HepG2 and H4IIE) and breast cancer (MCF-7 and BT549) cells, we showed that Bal induced apoptosis in liver cancer cells via increasing the expression of pro-apoptotic markers involved in mitochondrial mediated (caspase-3, Bax, Bid, Bad, and p53), death receptor mediated (caspase-3 and -8) and endoplasmic reticulum (ER)-stress–mediated (GRP78 and CHOP) apoptotic pathways, with no effect observed in the expression of anti-apoptotic genes (Bcl-2 and Bcl-XL). However, in breast cancer cells, Bal increased the expression of pro-apoptotic markers and also simultaneously decreased the expression of anti-apoptotic genes, triggering mitochondrial mediated and death receptor mediated apoptosis. Interestingly, Bal did not activate the ER-stress–mediated pathway in breast cancer cells, suggesting diverse mechanisms of apoptosis induction in different cancer cells (Ajji et al., 2017).

Bal and flavonoids (Nar, Nir, and Qu) appear to exert antitumor effects and induce apoptosis through similar mechanisms in liver (HepG2) and breast (MCF-7) cancer cells, therefore, we hypothesized that Bal-flavonoid (Nar, Nir, and Qu) combinations might have additive apoptotic effects on these cells. Thus, in this study, we examined the effect of co-treatment of Bal and three flavonoids, namely Nar, Nir, and Qu, on HepG2 and MCF-7 cells.

## Materials and Methods

### Chemicals, Reagents, and Kits

Nar (purity ≥ 95%), Nir (purity ≥ 95%), and Qu (purity ≥ 95%), trypsin, *in vitro* toxicology assay kit, Caspase-3 assay kit and Caspase-8 assay kit were procured from Sigma-Aldrich (USA). RPMI and DMEM media, and FBS were purchased from Life Technologies Corporation (California, USA). Total RNA isolation kit was purchased from Qiagen (Hilden, Germany). CM Sepharose and Superdex 75 for Balsamin purification were procured from GE Healthcare (Buckinghamshire, UK). Reagents used for electrophoresis were purchased from Bio Rad Laboratories (California, USA). All other reagents were purchased from Sigma-Aldrich (USA) and were of analytical grade.

### Purification of Balsamin

Balsamin was purified from *Momordica balsamina* seeds based on our earlier published method (Kaur et al., 2012). The seeds were powdered and homogenized in 50 ml of 150 mM NaCl. The slurry obtained was centrifuged at 12,000 g for 20 min at 4°C. Following centrifugation, the proteins were precipitated from the supernatant with the addition of ammonium sulphate. The precipitate obtained after centrifugation was dissolved in 10 mM phosphate buffer (PB), pH 6.5, and dialysed against the same buffer. Following equilibration of a CM-Sepharose column with PB, pH 6.5, the dialysed sample was loaded onto the column and elution of unbound protein was carried out with PB, pH 6.5. The bound proteins were eluted with 0.1–0.4 M NaCl in PB, pH 6.5. The peak fractions containing the protein of interest (analyzed by SDS-PAGE) were pooled, concentrated and loaded onto a Superdex 75 column equilibrated with PB, pH 6.5. The eluted fractions with the desired protein were pooled, concentrated, and further used for this study. The purified Bal was characterized by SDS-PAGE and protein concentration was determined by the Bradford method (Kaur et al., 2012).

### Cell Lines and Cell Culture

Liver (HepG2) and breast (MCF-7) cancer cells lines were investigated. These cell lines were procured from the American Type Culture Collection (Rockville, MD, US). HepG2 and MCF-7 cells were maintained in RPMI and DMEM media supplemented with heat inactivated 10% FBS, respectively, in an environment of 5% CO_2_ at 37°C. These cells were proliferated in T-25 flasks until 80% confluence and split 1:10 and 1:4, separately, in culture dishes before experiments were performed.

### Treatment Groups

**Figure 2:**
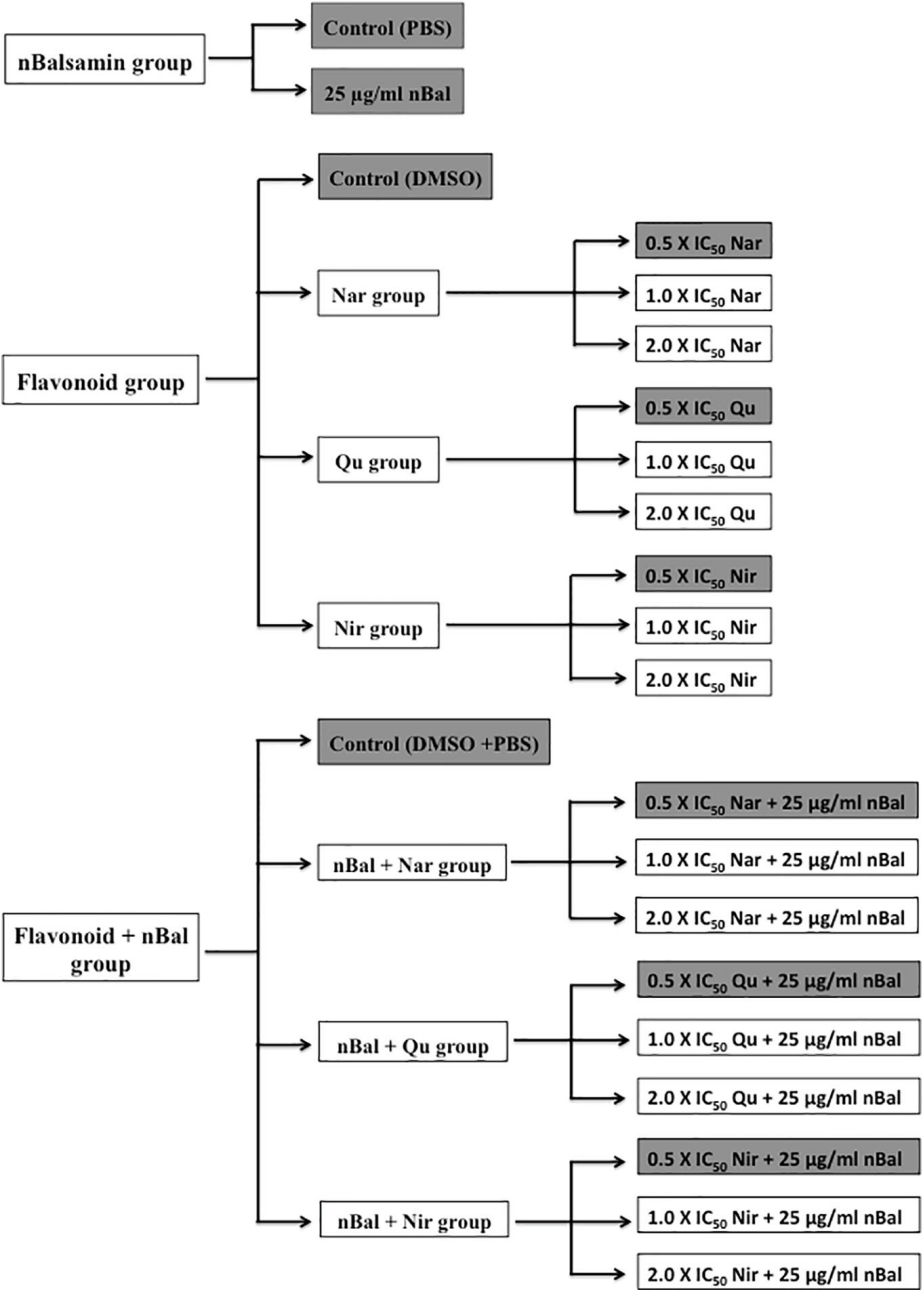
Treatment regime used for the study (IC_50_ values for Nar, Nir and Qu are 150 μM, 20 μM and 37 μM respectively).

For the cell viability assay, the treatment regimes given in **Figure 2** were used. Based on empirical data, treatments highlighted in **Figure 2** were selected for all other assays.

### Cell Viability Assay

HepG2 and MCF-7 cells were seeded in 96 well plates in a seeding density of ~1 × 10^4^ cells/well for 24 h. After incubation, the cells were exposed to the treatment regimes given in **Figure 2** for 24 h. Cell viability was then analyzed using an *in vitro* toxicology assay kit. MTT reagent was added to each well and the cells were incubated for 4 h. The MTT formazon dye crystals formed during the experiment were dissolved in MTT solubilization solution and the absorbance was measured using a microplate reader (Bio-Rad, USA) at a wavelength of 570 nm. All experiments were performed three times and results were expressed as mean ± standard deviation.

### Morphology Assay

Hep G2 and MCF-7 cells were seeded in 6-well plates in a seeding density of (~1 × 10^5^ cells/well for 24 h. After 24 h, the cells were treated with the treatments highlighted in **Figure 2** for 48 h. Cell morphology was then examined using a light microscope.

### Caspase-3/-8 Activity Assay

HepG2 and MCF-7 cells were seeded in 24 well plates in a seeding density of 6 × 10^4^ cells/well) for 24 h. After 24 h, the cells were treated with treatments highlighted in **Figure 2** for 48 h. After collecting and lysing the cells; caspase-3 and caspase-8 activity was determined immediately. The activity of caspase-3 and caspase-8 in triplicate was determined using caspase-3 and caspase-8 colorimetric assay kits according to the manufacturer’s protocol. Briefly, 5 µl of cell lysate and 1X assay buffer were added to the 96-well plate followed by caspase-3 substrate (AcDEVD-pNA) and caspase-8 substrate (AcIETD-pNA) to each well, respectively. The plates were incubated at 37°C for 2 h. Finally, the absorbance of pNA catalyzed by caspase-3 and caspase-8, respectively were measured using microplate system at 405 nm. All experiments were performed three times and results were expressed as mean ± standard deviation.

### RNA Extraction and RT-PCR for Gene Expression Studies

HepG2 and MCF-7 cells were seeded in 24 well plates in a seeding density of 6 × 10^4^ cells/well for 24 h. After incubation, the cells were treated with the treatments highlighted in **Figure 2** for 48 h. After treatment, total RNA was extracted using a Total RNA isolation kit (Qiagen) according to the protocol described by the manufacturer.

Maxima H minus first strand cDNA synthesis kit was used to synthesize first strand complementary DNA (cDNA) from each RNA sample. RT-PCR was carried out at 50°C for 30 min, followed by 85°C for 5 min and 4°C for 5 min, using the following primers sets: Bid: 5’- GCT GTA TAG CTG CTT CCA GTG TA -3’ (forward), 5’- GCT ATC TTC CAG CCT GTC TTC TC -3’ (reverse); p53: 5’- CTG TCA TCT TCT GTC CCT TC -3’ (forward), 5’- TGG AAT CAA CCC ACA GCT GCA -3’ (reverse); Bax: 5’- CTG CAG AGG ATG ATT GCC G -3’ (forward), 5’- TGC CAC TCG GAA AAA GAC CT -3’ (reverse); Bad: 5’- GCA CAG CAA CGC AGA TGC -3’ (forward), 5’- AAG TTC CGA TCC CAC CAG G -3’ (reverse); Bcl2: 5’- GTG TGG AGA GCG TCA ACC G -3’ (forward), 5’- CCT CTG TTT GAT TTC TCC TGG CT -3’ (reverse); Bcl-XL: 5’- GAT CCC CAT GGC AGC AGT AAA GCA AG -3’ (forward), 5’- CCC CAT CCC GGA AGA GTT CAT TCA CT -3’ (reverse); GRP78: 5’- GGT GAC CTG GTA CTG CTT GAT G -3’ (forward), 5’- CCT TGG AAT CAG TTT GGT CAT G -3’ (reverse); CHOP: 5’- TGC TTC TCT GGC TTG GCT GAC -3’ (forward), 5’- CCA AGG GAG AAC CAG GAA ACG G -3’ (reverse) (see **Table 1**).

**Table 1:** Primers used for RT-PCR.

Gene	Forward primer (5'-3')	Reverse primer (5'-3')
**Bid**	GCTGTATAGCTGCTTCCAGTGTA	GCTATCTTCCAGCCTGTCTTCTC
**p53**	CTGTCATCTTCTGTCCCTTC	TGGAATCAACCCACAGCTGCA
**Bax**	CTGCAGAGGATGATTGCCG	TGCCACTCGGAAAAAGACCT
**Bad**	GCACAGCAACGCAGATGC	AAGTTCCGATCCCACCAGG
**Bcl2**	GTGTGGAGAGCGTCAACCG	CCTCTGTTTGATTTCTCCTGGCT
**Bcl-XL**	GATCCCCATGGCAGCAGTAAAGCAAG	CCCCATCCCGGAAGAGTTCATTCACT
**GRP78**	GGTGACCTGGTACTGCTTGATG	CCTTGGAATCAGTTTGGTCATG
**CHOP**	TGCTTCTCTGGCTTGGCTGAC	CCAAGGGAGAACCAGGAAACGG

PCR was carried out in a reaction mixture of 10 µl under following conditions: 95°C for 7 min, 40 cycles at 95°C for 30 s and 60°C for 1 min, 60°C for 60 s, 55°C–95°C (hold time: 1 s, temperature increment after hold: 0.2°C), and 20°C for 10 s. All experiments were performed three times and results were expressed as mean ± standard deviation.

### Statistical Analysis

Data was analyzed using SPSS Statistics software version 22.0. Data was presented as the mean ± SD for at least three independently performed experiments unless otherwise stated. The non-parametric test, Kruskal-Wallis H test, also known as one-way ANOVA on ranks, was used to compare the difference between two or more groups. Mann-Whitney U test was used to compare the difference between two groups. Probability values with p < 0.05 were considered to be statistically significant.

## Results

### Growth Inhibitory Effects of Bal Combined With Flavonoids on Hepg2 and Mcf-7 Cells

**Figure 3:**
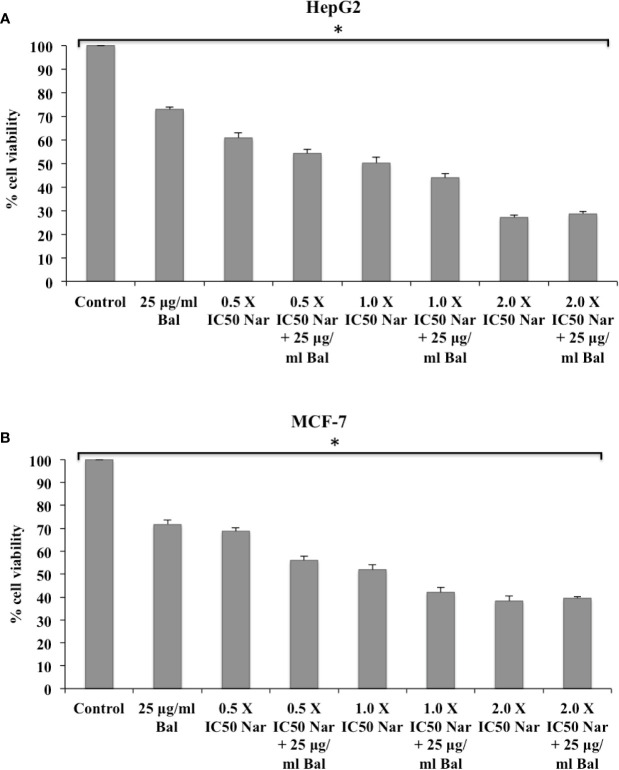
Effect of Bal and Nar on the viability of HepG2 **(A)** and MCF-7 **(B)** cells compared with Bal. All values are mean ± SD. SD, standard deviation; n = 3; *P < 0.05, Kruskal-Wallis H test.

We first determined the effect of co-treatment of Bal (25 µg/ml) and flavonoids (Nar, Nir, or Qu) on the viability of HepG2 and MCF-7 cells using an *in vitro* toxicology assay kit. As shown in **Figures 3A, B**, the combination of Bal with low dose (0.5 × IC_50_ dose) of Nar appeared to inhibit the viability of HepG2 and MCF-7 cells compared to the cells treated with Bal and Nar alone. However, when the concentration of Nar (1.0 × IC_50_ and 2.0 × IC_50_) was increased in combination with Bal, no significant difference between the Bal-Nar and Nar treated groups was observed, suggesting that 0.5 × IC_50_ of Nar with Bal could be the most effective dose combination (**Figures 3A, B**).

**Figure 4:**
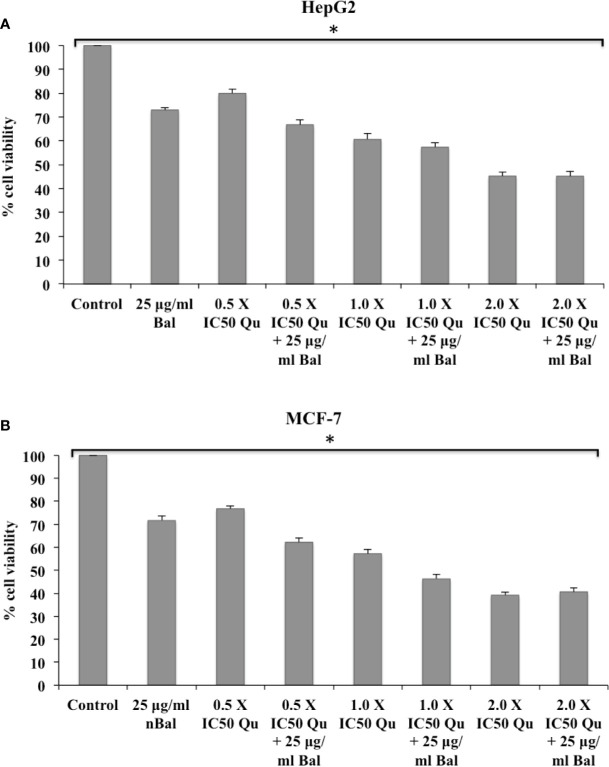
Effect of Bal and Qu on the viability of HepG2 **(A)** and MCF-7 **(B)** cells compared with Bal. All values are mean ± SD. SD, standard deviation; n = 3; *P < 0.05, Kruskal-Wallis H test.

**Figure 5:**
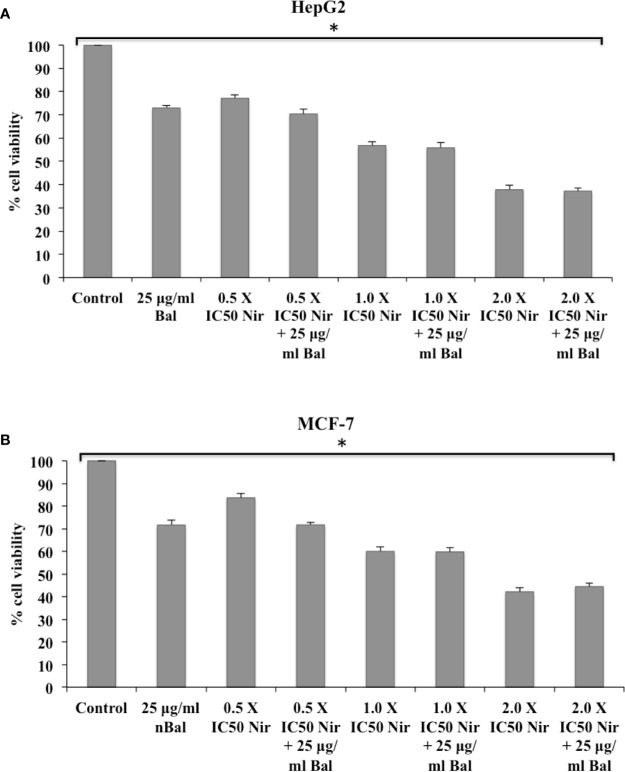
Effect of Bal and Nir on the viability of HepG2 **(A)** and MCF-7 **(B)** cells compared with Bal. All values are mean ± SD. SD, standard deviation; n = 3; *P < 0.05, Kruskal-Wallis H test.

Further, the trend appeared to be similar when HepG2 and MCF-7 were treated with Bal-Qu combinations (**Figures 4A, B**). However, when HepG2 and MCF-7 cell were co-treated with low dose (0.5 × IC_50_) of Nir and Bal, the cell viability tended to decrease slightly compared to singly treated groups, however, the effect was not as prominent as observed with low dose (0.5 × IC_50_) of Nar or Qu with Bal. Further, when the concentration of Nir (1.0 × IC_50_ and 2.0 × IC_50_) was increased in combination with Bal, no significant difference was observed in the groups treated with Bal-Nir combinations and Nir alone (**Figures 5A, B**). These results suggest that flavonoids (Nar, Nir, and Qu) could increase the anti-proliferative effects of Bal on HepG2 and MCF-7 cells, with low dose (0.5 × IC_50_) of flavonoids (Nar, Nir, and Qu) found to be the most effective dose in combination with Bal. Therefore, we selected this flavonoid dose in combination with Bal for further study.

### Effect of Bal-Flavonoid Combination on the Morphology of HepG2 and MCF-7 Cells

**Figure 6:**
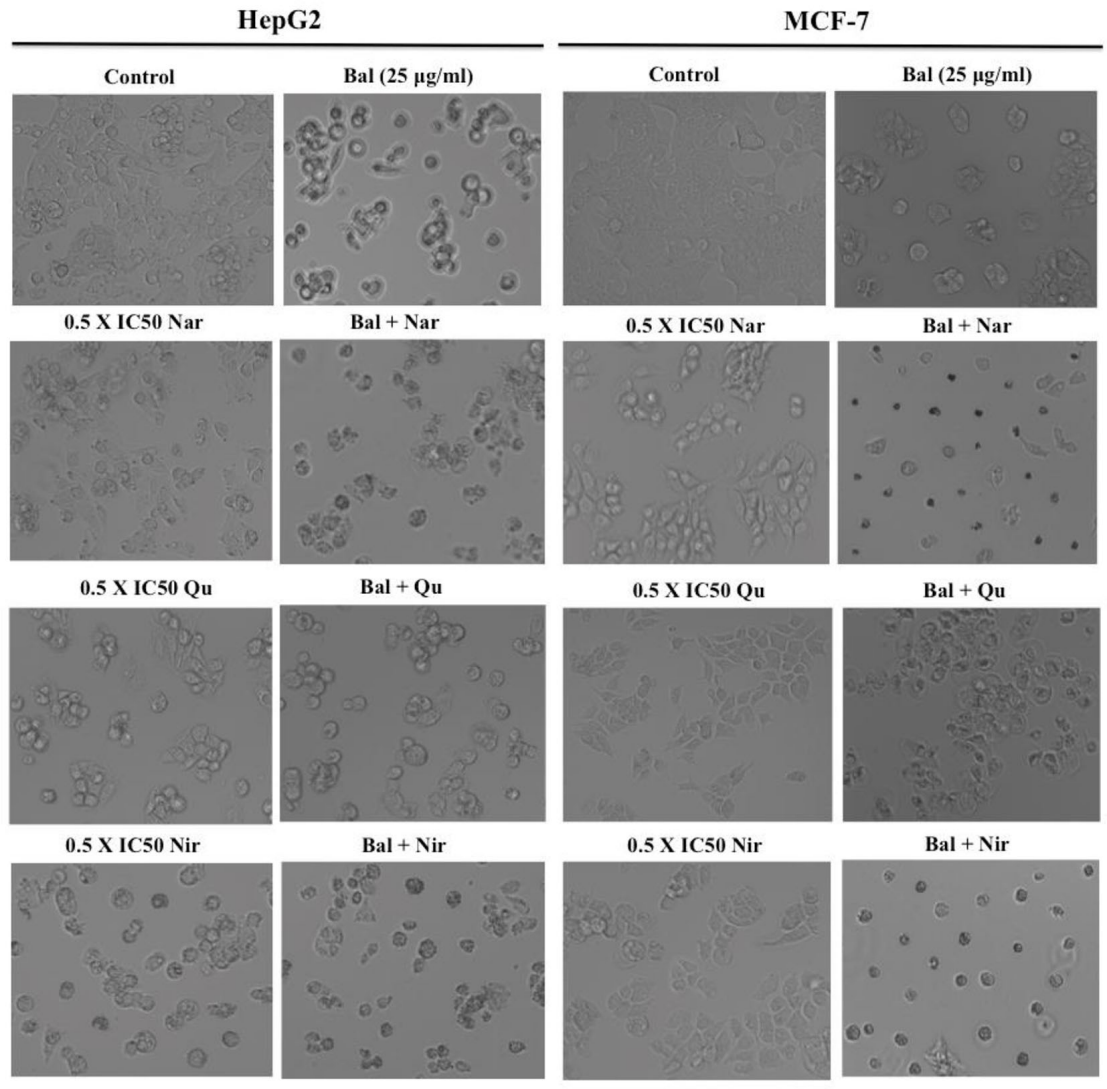
Morphology of HepG2 and MCF-7 cells treated with Bal-Nar, Bal-Qu and Bal-Nir compared with Bal, Nar, Qu and Nir alone.

We next investigated if flavonoids (Nar, Nir, or Qu) could increase Bal induced apoptotic effects in HepG2 and MCF7 cells. For this, we examined the morphology of HepG2 and MCF-7 cells treated with combinations of Bal and Nar, Nir, or Qu, and compounds alone under bright field microscopy. The results showed that apoptotic morphological changes, such as loss in cell connections, cell shrinkage, cell surface detachment, increased cytoplasmic density and more dead cells appeared to be more prominent in cells treated with Bal-flavonoid (Nar, Nir, or Qu) combinations compared to single treatments (**Figure 6**).

### Caspase-3 and -8 Activity of Bal Combined With Nar, Nir, or Qu in HepG2 and MCF-7 Cells

**Figure 7:**
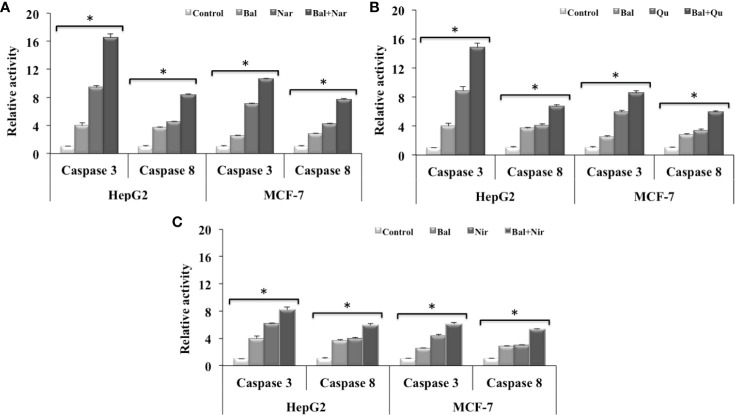
Effect of Bal-Nar **(A)**, Bal-Qu **(B)** and Bal-Nir **(C)** on caspase-3 and -8 activity compared with Bal in HepG2 and MCF-7 cells. All values are mean ± SD. SD, standard deviation; n = 3; *P < 0.05, Kruskal-Wallis H test.

We further decided to evaluate whether flavonoids (Nar, Nir, and Qu) increase caspase-mediated apoptotic effects in Bal treated HepG2 and MCF-7 cells. As shown in **Figure 7A**, Bal-Nar treatment tended to increase and appeared to have an additive effect on caspase-3 and -8 activity in HepG2 and MCF-7 cells compared to Bal and Nar alone. Further, the trend appeared to be similar in HepG2 and MCF-7 cells treated with the Bal-Qu combination (**Figure 7B**). However, when HepG2 and MCF-7 cells were treated with the Bal-Nir combination, the caspase-3 and -8 activity tended to increase as compared to Nir and Bal treatment alone, however, did not appear to have an additive effect (**Figure 7C**).

### Effect of Bal-Flavonoid Combinations on the Expression of Various Apoptotic Genes Involved in the Mitochondrial Cell Death Pathway

**Figure 8:**
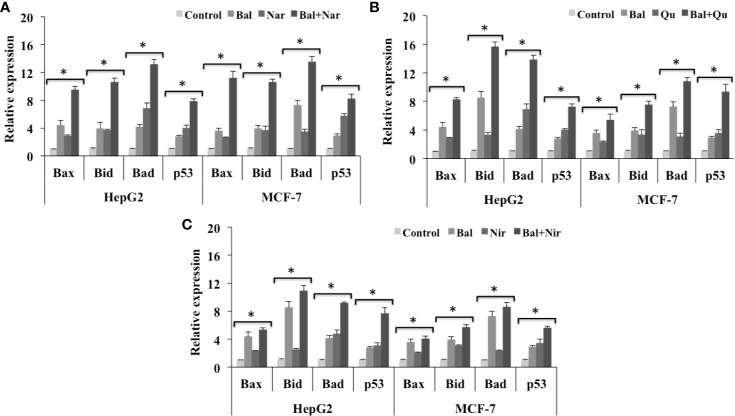
Effect of Bal-Nar **(A)**, Bal-Qu **(B)** and Bal-Nir **(C)** on the expression of pro-apoptotic genes, Bax, Bid, Bad and p53, compared with Bal in HepG2 and MCF-7 cells. All values are mean ± SD. SD, standard deviation; n = 3; *P < 0.05, Kruskal-Wallis H test.

We next studied if these flavonoids could increase Bal-induced mitochondrial apoptotic effects in HepG2 and MCF-7 cells. The qRT-PCR results showed that Bal-Nar treatment tended to increase and appeared to have an additive effect on the expression of *Bax*, *Bid*, *Bad*, and *p53* compared to Bal treatment alone in HepG2 and MCF-7 cells. However, no significant difference was observed between the groups (**Figure 8A**). Further, the trend appeared to be similar in HepG2 and MCF-7 cells treated with the Bal-Qu combination (**Figure 8B**), suggesting that Nar and Qu increase Bal-induced apoptotic effect in HepG2 and MCF-7 cells.

In addition, the Bal-Nir combination appeared to increase the expression of *Bax*, *Bid*, *Bad* and *p53* as compared Bal treatment alone in HepG2 and MCF-7 cells. However, the effect did not appear to be pronounced and additive compared to Bal-Nar and Bal-Qu treatment (**Figure 8C**), suggesting that the Bal-Nar and Bal-Qu combinations could be considered more effective than the Bal-Nir combination.

**Figure 9:**
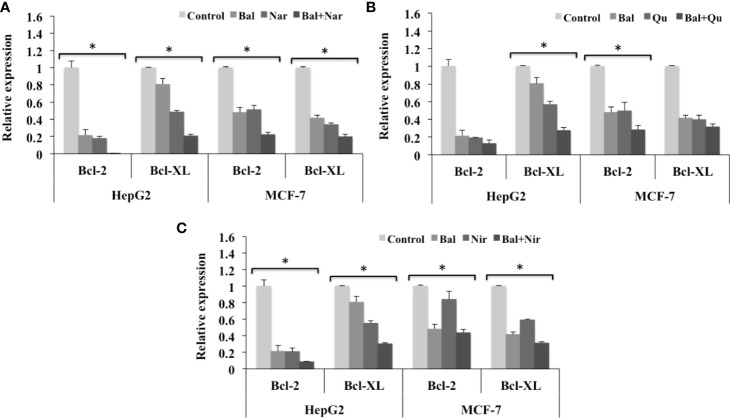
Effect of Bal-Nar **(A)**, Bal-Qu **(B)** and Bal-Nir **(C)** on the expression of anti-apoptotic genes, Bcl-2 and Bcl-XL, compared with Bal in HepG2 and MCF-7 cells. All values are mean ± SD. SD, standard deviation; n = 3; *P < 0.05, Kruskal-Wallis H test.

Further, we also evaluated the expression of anti-apoptotic genes (*Bcl-2* and *Bcl-XL*) in HepG2 and MCF-7 cells treated with Bal-flavonoid (Nar, Nir, or Qu) combinations. The qRT-PCR results showed that Bal-Nar treatment tended to decrease the expression of *Bcl-2* and *Bcl-XL* compared to Bal and Nar treatment alone in HepG2 and MCF-7 cells, indicating a possible effect. However, no significant difference was observed between the groups (**Figure 9A**). Likewise, the trend appeared to similar, but less pronounced in HepG2 and MCF-7 cells treated with Bal-Qu and Bal-Nir combinations (**Figures 9B, C**).

### Effect of Bal-Flavonoid Treatment on the Expression of Genes Involved in Endoplasmic Reticulum-Stress–Mediated Apoptosis

To investigate whether Bal-flavonoid treatment could increase Bal-induced ER stress in HepG2 cells and activate this pathway in breast cancer (MCF-7) cells, we evaluated the expression of *CHOP* and *GRP78* in Bal-flavonoid (Nar, Nir, and Qu) treated HepG2 and MCF-7 cells.

**Figure 10:**
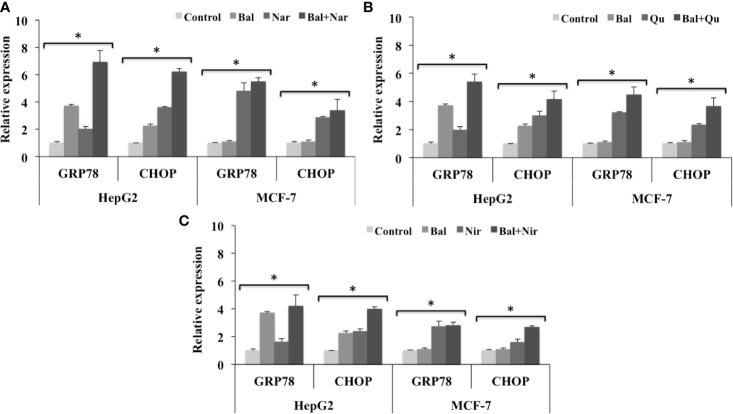
Effect of Bal-Nar **(A)**, Bal-Qu **(B)** and Bal-Nir **(C)** on the expression of gene, GRP78 and CHOP, involved in ER-stress mediated apoptosis, compared with Bal in HepG2 and MCF-7 cells. All values are mean ± SD. SD, standard deviation; n = 3; *P < 0.05, Kruskal-Wallis H test.

The qRT-PCR results showed that the expression of *GRP78* and *CHOP* tended to increase in HepG2 cells treated with Bal-Nar (**Figure 10A**), Bal-Qu (**Figure 10B**), and Bal-Nir (**Figure 10C**) combinations compared to Bal and flavonoids (Nar, Nir, and Qu) treatment alone, indicating a possible additive effect of co-treatment in HepG2 and MCF-7 cells. In the case of MCF-7 cells, Bal treatment alone did not increase the expression of *GRP78* and *CHOP*. However, when Bal was combined with the flavonoids (Nar, Qu, and Nir), the expression tended to increase with respect to Bal alone, but did not increase significantly with respect to flavonoid (Nar, Qu, and Nir) treatment alone, suggesting that the effect was entirely due to the presence of the flavonoid (Nar, Qu, or Nir) in the Bal-flavonoid combinations (**Figures 10A–C**).

## Discussion

The relationship between diet and cancer has been implicated in number of epidemiological studies (Ross, 2010). Vegetables, fruits, and cereals contain flavonoids that have antioxidant, anti-inflammatory and anticancer properties and Nar, Nir, and Qu are three such flavonoids that have been explored for their anti-proliferative effects toward various cancer cells. Nar, a natural flavone exhibits anti-tumor activity toward various tumor cell types including hepatoma (HepG2), breast cancer (MCF-7, HTB26 and HTB132), leukemia (K562), human epidermoid carcinoma (A431), colorectal cancer (SW1116 and SW837), and glioma cells (C6) (Harmon and Patel, 2004; Sabarinathan et al., 2011; Arul and Subramanian, 2013; Ahamad et al., 2014; Abaza et al., 2015; Li et al., 2015). Nir exerts antiproliferative effects on cancer cells such as breast cancer (MDA-MB-231 and BT549), liver cancer (HepG2), bladder cancer (5637), melanoma (A375), and glioblastoma (U87MG) (Kim et al., 2008; Guo et al., 2016; Li et al., 2016). Qu possesses antitumor activity against liver cancer (HepG2), breast cancer (MCF-7), leukemia (HL-60) and prostate cancer (DU-145 and PC-3) cells (Nair et al., 2004; Niu et al., 2011; Deng et al., 2013; Zhao et al., 2014).

Nar caused cell death via cytotoxic, genotoxic and apotosis effect on various cancer cell lines including HT-29, MCF-7 and PC-12 through the production of ROS and DNA damage in a dose dependent manner. However, the effects were found to be nominal in case of normal cell line, L-929 as the IC_50_ value of Nar for L-929 cell line could not be dertemined since the value was out of range (Kocyigit et el., 2016). Qu induced cytotoxicity in a dose dependent manner in leukemic (CEM and K562) and breast cancer (T47D and EAC) cells through DNA fragmentation, cell cycle arrest and activating intrinsic pathway of apoptosis. “However, normal cells dervived from human embryonic kidney (293T) as well as mouse embryonic fibroblast (MEF-1) were found to be insensitive to quercetin (Srivastava et al., 2016)” at similar concentrations able to induce death in carcinogenic cells (different works have already published this result).

Several studies have reported that flavonoids with antioxidant properties synergize the effect of chemotherapeutic drugs (Lewandowska et al., 2014). Qu increased cisplatin-induced apoptosis in human laryngeal cancer (Hep-2) cells (Kuhar et al., 2007). 4T1 breast tumor mice treated with Qu and doxorubicin demonstrated inhibition of tumor growth and reduction of doxorubicin side effects, leading to prolonged survival of the mice (Du et al., 2010). Qu enhanced the antitumor effect of trichostatin-A, a novel anticancer drug, in human lung cancer cells through upregulation of p53 expression (Chan et al., 2013).

Our previous studies demonstrated that Bal, a type I RIP isolated from *Momordica balsamina*, exhibits inhibitory effects toward HIV-1 replication (Kaur et al., 2013) and breast cancer cells, highlighting its potential as a therapeutic agent. It has previously been reported that Bal-induced apoptosis involved increases in caspase-3 and caspase-8 activity, cell cycle arrest at G-/S- phase, upregulation of Bax, Bid, and Bad, and downregulation of BCL-2 and BCL-XL (Ajji et al., 2017).

Since flavonoids (Nar, Nir, and Qu) and Bal exerts anticancer effects, therefore the present study investigated the apoptotic effects of Bal in the presence of three flavonoids, namely Nar, Nir, and Qu, on liver (HepG2) and breast cancer (MCF-7) cells. Co-treatment with Bal (25 µg/ml) and flavonoids (Nar, Nir, or Qu) enhanced the anti-proliferative effects of Bal on HepG2 and MCF-7 cells where low dose (0.5 × IC_50_) of flavonoids (Nar, Nir, and Qu) in combination with Bal was found to be an effective dose as compared to other two doses (1.0 × IC_50_ and 2.0 × IC_50_) of Nar, Nir, or Qu, and henceforth the lowest dose (0.5 × IC_50_) of Nar, Nir, and Qu was selected in combination with Bal for further study. These doses were selected based on the empirical data; however, further investigations would be required to evaluate appropriate doses of Bal and flavonoids for alleviating liver and breast cancer in clinical trials

Flavonoids, such as Nar, Nir, and Qu are known to induce apoptosis in various cancer cells by regulating the expression of apoptotic markers (Arul and Subramanian, 2013; Banjerdpongchai et al., 2016). Apoptosis is a programmed and often energy dependent process that involves activation of caspases, a group of cysteine proteases, and a complex cascade of signals (Elmore, 2007). Flavonoids such as Nar, Nir, and Qu have been reported to activate caspase dependent apoptosis in cancer cells (Ramos, 2007). Nar and Nir induced apoptosis in HepG2 cells via activating caspase-8 and caspase-9 mediated cell death pathways (Arul and Subramanian, 2013; Banjerdpongchai et al., 2016). Qu induced cell death in glioblastoma U373MG cells through proteolytic activation of caspase-3 and caspase-7, a decrease in mitochondrial membrane potential and increases in caspase-3 and caspase-9 activities (Kim et al., 2013). Our previous studies have demonstrated that Bal also induces apoptosis by increasing the activity of caspase-3 and -8 in breast cancer cells (Ajji et al., 2017). In this study, Bal in combination with flavonoids (Nar, Nir, and Qu) had an additive effect on caspase-3 and -8 activity in HepG2 and MCF-7 cells compared to Bal and flavonoids (Nar, Nir, and Qu) alone. Out of three combinations, Bal-Nar and Bal-Qu appeared to have a more prominent effect compared to Bal-Nir combination.

Studies have shown that apoptosis is also regulated by the Bcl-2 family of pro-apoptotic and anti-apoptotic genes. Flavonoids, such as Nar, Nir, and Qu, have been reported to trigger apoptosis via regulating the expression of pro-apoptotic and anti-apoptotic genes involved in the mitochondrial cell death pathway (Ramos, 2008). Nar isolated from *Thymus vulgaris* upregulated pro-apoptotic markers, p18, p19, p21, Bax and Bak, and downregulated anti-apoptotic markers, Cdk4, Cdk6, Cdk7, and Bcl-2 in human colorectal and breast cancer cells leading to apoptosis (Abaza et al., 2015). Qu induced apoptosis in HepG2 hepatoma cells by decreasing the Bcl-XL:Bcl-XS ratio, activating caspase-3 and -9 and increasing the translocation of Bax to the mitochondrial membrane, leading to mitochondrial mediated apoptosis (Granado-Serrano et al., 2006). Our previous studies demonstrated that Bal induces the mitochondrial cell death pathway in breast cancer cells by increasing the expression of pro-apoptotic genes, *Bax*, *Bid*, *Bad*, and *p53*, and decreasing the expression of anti-apoptotic genes, *Bcl-2* and *Bcl-XL* (Ajji et al., 2017). In this study, Bal- flavonoid (Nar, Nir, and Qu) treatment tended to increase and appeared to have an additive effect on the expression of *Bax*, *Bid*, *Bad*, and *p53* in HepG2 and MCF-7 cells compared to Bal and flavonoids (Nar, Nir, and Qu) treatment alone, where Bal-Nar and Bal-Qu appeared to have more pronounced and additive effect compared to Bal-Nir combination. Increase in activation of caspase-3 and -8, upregulation of pro-apoptotic genes (*Bax*, *Bid*, *Bad*, *p53*) and downregulation of anti-apoptotic genes (*Bcl-2* and *Bcl-XL*) with Bal-Nar, Bal-Nir and Bal-Qu treatment compared to Bal and flavonoid (Nar, Nir, or Qu) treatment alone in HepG2 and MCF-7 cells, suggested that these flavonoids increase Bal-induced mitochondrial mediated apoptosis with a possible additive effect in HepG2 and MCF-7 cells, which could be considered as a promising strategy to sensitize cells to Bal treatment.

The ER stress response has been reported to initiate apoptosis in various cancer cells, through three different pathways; (i) activation of *CHOP*, also known as DNA damage-inducible gene 153 (*GADD153*), (ii) activation of ER associated caspases and (iii) activation of c-Jun N- terminal kinase (JNK)-mediated cell death (G Johnson et al., 2011). Qu induced ER-stress–mediated apoptosis by increasing the expression of *ATF*, *GRP78* and *CHOP* in prostate cancer PC-3 cells (Liu et al., 2014). It has been reported that Qu pre-treatment elicits ER-stress to enhance cisplatin cytotoxicity in ovarian cancer cells, indicating that Qu has a potential to enhance the efficacy of cisplatin induced apoptosis in ovarian cancer cells (Yang et al., 2015). Our studies have shown that Bal does not activate ER-stress–mediated apoptosis in breast cancer (MCF-7 and BT549) cells (Ajji et al., 2017). However, co-treatment with Bal and flavonoids (Nar, Nir, and Qu) tended to increase and appeared to have additive effect on the expression of *GRP78* and *CHOP* in HepG2 cells compared to Bal and flavonoids (Nar, Nir, and Qu) treatment alone. However, in case of MCF-7 cells, Bal- flavonoid (Nar, Qu, or Nir) treatment tended to increase the expression *GRP78* and *CHOP* with respect to Bal alone, but did not increase significantly with respect to flavonoid (Nar, Qu, and Nir) treatment alone, indicating that the effect was entirely due to the presence of the flavonoid in the combined treatment. It is interesting to note that these flavonoids activate ER-stress–mediated apoptosis, an additional apoptotic pathway, in breast cancer MCF-7 cells that is not activated by Bal treatment alone.

A number of studies have reported the bioavailability of flavonoids such as Nar, Qu, and Nir in rats. The plasma concentration of Nar and Qu metabolites in rats receiving single meal containing 0.25% Nar and 0.2% Qu after 24 h was found to be 128 µM and 51 µM, respectively. The plasma concentration of Nar and Qu increased till 10-12 h after single feed and decreased thereafter indicating that these flavonoids are efficiently absorbed in rats (Manach et al., 1997; Felgines et al., 2000). Our results suggested that consumption of these flavonoids (Nar, Qu, and Nir) with Bal at selected doses might be beneficial to prevent and treat liver and breast cancer, thus advocating its use as a dietary supplement. It could be a promising therapeutic strategy to sensitize liver and breast cells to Bal treatment, thereby improving its effectiveness in cancer therapy. However, further investigations would be required to evaluate appropriate doses of Bal and flavonoids for alleviating liver and breast cancer in clinical trials.

## Conclusions

To the best of our knowledge, our study demonstrates for the first time that Bal in combination with flavonoids (Nar, Nir, and Qu) enhances apoptotic effects in HepG2 and MCF-7 cells through activation of caspase-3 and -8 and regulation of pro-apoptotic and anti-apoptotic genes. These results suggested that Bal with flavonoids (Nar, Qu, and Nir) could be a promising therapeutic strategy to reduce proliferation in breast and liver cancer cells and sensitize cells to Bal treatment. However, the study warrants further investigation to evaluate the efficacy and appropriate doses of Bal and flavonoids for alleviating liver and breast cancer in clinical trials.

## Data Availability Statement

The raw data supporting the conclusions of this article will be made available by the authors, without undue reservation.

## Author Contributions

PA conducted experiments, analyzed data, and wrote the manuscript. KW participated in the experimental planning, analyzed the data, and edited the manuscript. MP conceived the study, planned the experiments, analyzed the data, and edited the manuscript. All authors contributed to the article and approved the submitted version.

## Conflict of Interest

The authors declare that the research was conducted in the absence of any commercial or financial relationships that could be construed as a potential conflict of interest.
